# Surgical Management of Unilateral Soft Tissue Swelling around the Proximal Interphalangeal Joint in an Adolescent: A Case Report of Pachydermodactyly

**DOI:** 10.1155/2015/242078

**Published:** 2015-11-24

**Authors:** Naoki Kato, Mamoru Niitsu, Tetsuya Kawabe, Eiichi Arai, Keizo Fukumoto

**Affiliations:** ^1^Saitama Hand Surgery Institute, Saitama Seikeikai Hospital, 1721 Ishibashi, Higashimatsuyama City, Saitama 355-0072, Japan; ^2^Department of Radiology, Saitama Medical University, 38 Morohongo, Moroyamacho, Iruma, Saitama 350-0451, Japan; ^3^Department of Radiology, Saitama Medical Center, Saitama Medical University, 1981 Kamoda, Kawagoe City, Saitama 350-8550, Japan; ^4^Department of Pathology, Saitama International Center, Saitama Medical University, 1397-1 Yamane, Hidaka City, Saitama 350-1298, Japan

## Abstract

Pachydermodactyly (PDD) is a rare and benign acquired form of digital fibromatosis, characterized by asymptomatic fusiform soft tissue hypertrophy of the lateral aspect of the proximal interphalangeal (PIP) joints of the fingers. The etiology of PDD remains unknown, but it usually affects healthy males around the age of puberty. It can be misdiagnosed as inflammatory rheumatic diseases, especially as juvenile chronic arthritis. Here, we report a case of PDD in an 18-year-old man who had progressive fusiform swelling of the PIP joint on his left middle finger. Although he had no pain or functional limitation of movement, he chose to undergo surgical resection of the lesion to obtain a conclusive diagnosis and to rectify the deformity's appearance. Histologically, the lesion was characterized by coarse fibrosis in the adipose tissue, peripheral nerve fibers, and eccrine glands; this is compatible with a diagnosis of PDD.

## 1. Introduction

Pachydermodactyly (PDD), from the Greek words pachy (thick), dermo (skin), and dactylos (finger), was first reported by Bazex et al. in 1973 [1]. PDD is a benign, asymptomatic thickening of the soft tissues that overlie the radial and ulnar aspects of the proximal interphalangeal (PIP) joints but does not produce any fundamental damage to the joint structures. The etiology of PDD remains unknown. However, some authors believe the cause to be repetitive, exogenous, minor traumas due to habitual or compulsive habits of rubbing of fingers [1–4]. The diagnosis of PDD is clinical and is supported by radiological studies and histopathologic examination. Psychological counseling is sometimes required for the patients with compulsive behavior. The number of medical reports of PDD is very limited, and, to the best of our knowledge, almost all the cases of PDD have been reported in the fields of dermatology or rheumatology. In the field of orthopedic surgery, only one case report treated with tranilast has been published recently [5]. Here, we report a case of successful surgical management of PDD in an 18-year-old man who showed progressive lateral swelling of the PIP joint in the unilateral middle finger.

## 2. Case Report

**Figure 1:**
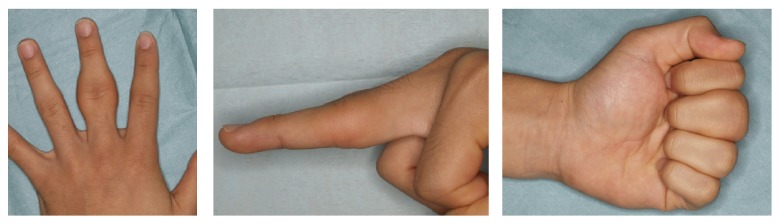
There was diffuse thickening, predominantly, of the radial and ulnar aspects of the PIP joint.

**Figure 2:**
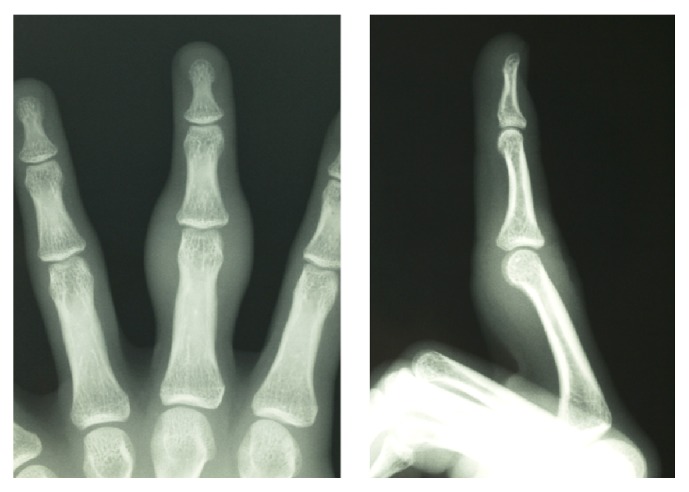
Fusiform soft tissue swelling without joint space narrowing was observed in the PIP joint of the middle finger. There were no bony erosions or other structural abnormalities.

**Figure 3:**
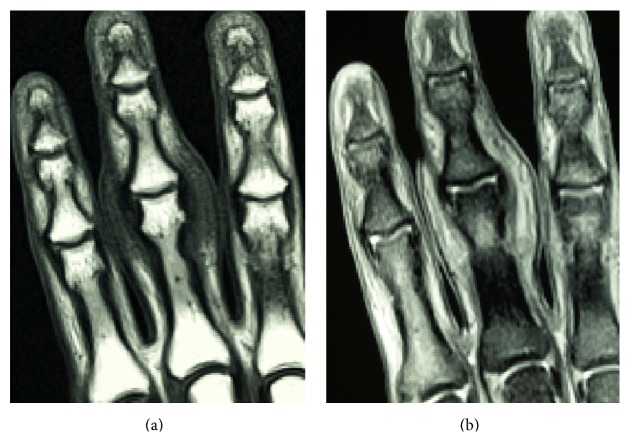
The lesion indicated homogenous isointensity to the muscle on a T1-weighted image (a). On the postcontrast fat-saturated T1-weighted image, the lesion was heterogeneously hyperintense, and no other abnormal enhancement was noted (b).

**Figure 4:**
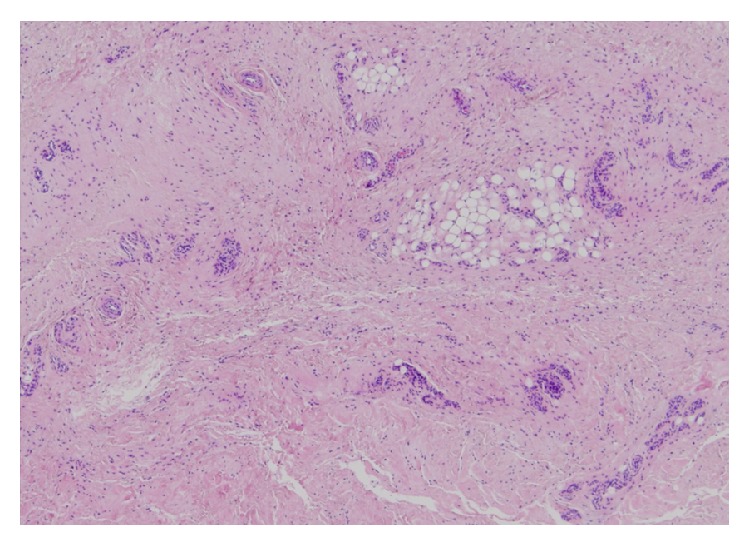
The lesion was characterized by coarse fibrosis with adipose tissue, peripheral nerve fibers, and eccrine glands.

**Figure 5:**
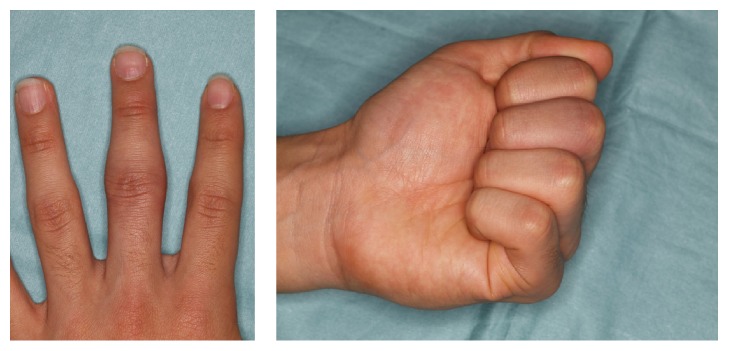
At the latest follow-up, no recurrence was observed in his middle finger.

An 18-year-old man presented at our hospital with asymptomatic, gradually increasing fusiform swelling of the PIP joint in his left middle finger. He was right-handed. Over the past 5 years, he had undergone examinations by various specialists without a convincing diagnosis being made. On physical examination, there was diffuse thickening, predominantly, of the radial and ulnar aspects of the PIP joint, without any skin pigmentation or neurological deficits (Figure 1). He had no pain or functional limitation of movement. Based on the clinical history, psychiatric disorders and emotional distress were excluded. No repetitive movement was noted. His family history was noncontributory. There were no signs of infection or inflammation in the joint involved, and there were no other joint or systemic symptoms. The results of laboratory investigations, including a complete blood count, liver/renal function tests, erythrocyte sedimentation rate test, C-reactive protein test, and rheumatoid factor test, were within the normal range or negative. Plain radiographs of the hands revealed fusiform soft tissue swelling without joint space narrowing in the PIP joint of the middle finger. There were no bony erosions or other structural abnormalities (Figure 2). Magnetic resonance imaging (MRI) of the hand revealed that the soft tissue swelling extended circumferentially and longitudinally over the PIP joints without joint effusion or tenosynovial effusion. The lesion indicated homogenous isointensity to the muscle on a T1-weighted image. On the postcontrast fat-saturated T1-weighted image, the lesion was heterogeneously hyperintense, and no other abnormal enhancement was noted. No fresh or chronic hemorrhagic change was demonstrated (Figure 3). Although he had no pain or functional limitation of movement, he chose to undergo surgical resection of the lesion to obtain a conclusive diagnosis and to rectify the deformity's appearance. Surgery was performed under general anesthesia. A volar Brunner incision was made from the distal interphalangeal crease to the palmar digital crease, and care was taken to protect the bilateral neurovascular bundles. Subcutaneous hypertrophic soft tissue was excised bilaterally to reduce the tissue volume. After excision, redundant skin was trimmed and the skin was closed by simple reefing. Gross examination revealed that the subcutaneous soft tissue lesion included skin and fibroadipose tissue. Histopathologically, the lesion was characterized by coarse fibrosis with adipose tissue, peripheral nerve fibers, and eccrine glands; this is compatible with a diagnosis of PDD (Figure 4). At the latest follow-up, 3 years postoperatively, no recurrence was observed in his middle finger, and he was very satisfied with the outcome, both cosmetic and functional ones (Figure 5).

## 3. Discussion

PDD has been recognized as a distinct clinical entity because of its typical presentation of asymptomatic thickening of the soft tissues that overlie the radial and ulnar aspects of the PIP joints without any fundamental damage to the joint structures [6]. The distal interphalangeal (DIP) joints and other joints are not usually affected. PDD mostly occurs in young healthy people at a mean age of 21.2 years, although it has been associated with Dupuytren contracture, Asperger syndrome, carpal tunnel syndrome, and tuberous sclerosis [7]. Because of the benign characteristics of PDD, most patients do not require therapy. However, some patients prefer to undergo surgical treatment to rectify the deformity's appearance, as in the present case. We performed surgical resection via the volar approach, and it was effective both cosmetically and functionally. This is the first report showing that surgical intervention is effective for PDD. We consider that a volar Brunner incision is useful to excise hypertrophic soft tissue and redundant skin bilaterally. It is also useful to protect the bilateral neurovascular bundles and to close the skin by simple reefing. However, care must be taken not to impair blood supply to the skin to be closed by excessive excision of the subcutaneous tissue. The diagnosis of PDD is made clinically and is supported by radiological and histological studies. Radiography, ultrasonography, and MRI show soft tissue thickening but no abnormalities of the joint space or bones [6]. Histologically, PDD is characterized by orthokeratotic or parakeratotic hyperkeratosis, acanthosis, and thickening of the dermis owing to an increased amount of fibroblasts and collagen types III and V; this differs from the collagen profile of normal skin [8]. Electron microscopy has demonstrated an increased number of thin, collagen fibers [9]. There is usually minimal or no inflammation. Our patient showed the typical clinical manifestations as well as normal radiologic and histological findings. The etiology of PDD is still unknown. However, some authors believe the cause to be repetitive, exogenous, minor traumas due to habitual or compulsive habits of rubbing of fingers [1–4]. In addition, occupational repetitive movement of fingers can be a cause of PDD [10]. The differential diagnosis includes secondary digital changes due to various disorders, such as pseudoknuckle pads, which is a term that has been used to describe the callosity that develops after repeated skin irritation. The distinction between pseudoknuckle pads and PDD is the location of skin swelling; PDD involves predominantly the lateral aspect of the PIP joint, whereas pseudoknuckle pads involve the dorsal surface [11]. Other differential diagnoses are as follows: foreign body granuloma, chewing pads, collagenous plaques of the hands, juvenile digital fibromatosis, progressive nodular fibrosis of the skin, thyroid disease, acromegaly, and so forth [3]. In addition, all arthropathies involving the PIP joints should be considered. PDD can be misdiagnosed as an inflammatory rheumatic disease, especially juvenile idiopathic arthritis. Such misdiagnoses may lead to needless investigations and inappropriate treatment [12], and therefore we consider it important for orthopedic surgeons to immediately recognize and diagnose PDD.

## References

[1] Bazex A., Dupré A., Teillard J. (1973). Pachydermie digitale des premières phalanges par hyperplasie conjonctive dermique et aplasie hypodermique. *Bulletin de la Société Française de Dermatologie et de Syphiligraphie*.

[2] Pereira J. M., Netto Pereira F. C., Netto Pereira V. C. (2004). Interphalangeal pads on pachydermodactyly. *Anais Brasileiros de Dermatologia*.

[3] Beltraminelli H., Itin P. (2009). Pachydermodactyly—just a sign of emotional distress. *European Journal of Dermatology*.

[4] Dupin N., Gautier M.-S., Rabary G., Auffret N., Beltzer-Garelly E., Binet O. (1994). Pachydermodactyly. *Annales de Dermatologie et de Venereologie*.

[5] Higuchi C., Tomita T., Yoshikawa H. (2014). Pachydermodactyly treated with tranilast in a young girl. *Case Reports in Orthopedics*.

[6] Castori M., Paradisi A. (2011). Pachydermodactyly with mild features of heritable connective tissue disorder and no sign of emotional distress. *Clinical and Experimental Dermatology*.

[7] Bardazzi F., Neri I., Raone B., Patrizi A. (1998). Pachydermodactyly: seven new cases. *Annales de Dermatologie et de Venereologie*.

[8] Ye S., Chen S.-L., Dong Y.-Q., Lin F., Guo Q., Bao C.-D. (2005). Pachydermodactyly: six new cases from China. *Journal of Clinical Rheumatology*.

[9] Kim T.-H., Cho Y.-H., Park H.-B. (1996). Two cases of pachydermodactyly. *Journal of Dermatology*.

[10] Sagransky M. J., Pichardo-Geisinger R. O., Muñoz-Ali D., Feldman S. R., Mora D. C., Quandt S. A. (2012). Pachydermodactyly from repetitive motion in poultry processing workers: a report of 2 cases. *Archives of Dermatology*.

[11] Vale L. R. G., Coeli F. R., Michalany N., Hassun K. M., Porro A. M. (2009). Transgrediens pachydermodactyly: report of a case. *Anais Brasileiros de Dermatologia*.

[12] Fathalla B. M., Goldsmith D. P. (2009). Pachydermatodactyly mimics polyarticular juvenile idiopathic arthritis. *Journal of Pediatrics*.

